# Perturbations of PIP3 signalling trigger a global remodelling of mRNA landscape and reveal a transcriptional feedback loop

**DOI:** 10.1093/nar/gkv1015

**Published:** 2015-10-12

**Authors:** Vladimir Yu. Kiselev, Veronique Juvin, Mouhannad Malek, Nicholas Luscombe, Phillip Hawkins, Nicolas Le Novère, Len Stephens

**Affiliations:** 1 Babraham Institute, Babraham Research Campus, Cambridge, CB22 3AT, UK; 2 London Research Institute, London, WC2A 3LY, UK; 3 EMBL-European Bioinformatics Institute, Hinxton, CB10 1SD, UK

## Abstract

PIP3 is synthesized by the Class I PI3Ks and regulates complex cell responses, such as growth and migration. Signals that drive long-term reshaping of cell phenotypes are difficult to resolve because of complex feedback networks that operate over extended times. PIP_3_-dependent modulation of mRNA accumulation is clearly important in this process but is poorly understood. We have quantified the genome-wide mRNA-landscape of non-transformed, breast epithelium-derived MCF10a cells and its response to acute regulation by EGF, in the presence or absence of a PI3Kα inhibitor, compare it to chronic activation of PI3K signalling by cancer-relevant mutations (isogenic cells expressing an oncomutant PI3Kα allele or lacking the PIP_3_-phosphatase/tumour-suppressor, PTEN). Our results show that whilst many mRNAs are changed by long-term genetic perturbation of PIP3 signalling (‘butterfly effect’), a much smaller number do so in a coherent fashion with the different PIP3 perturbations. This suggests a subset of more directly regulated mRNAs. We show that mRNAs respond differently to given aspects of PIP3 regulation. Some PIP3-sensitive mRNAs encode PI3K pathway components, thus suggesting a transcriptional feedback loop. We identify the transcription factor binding motifs SRF and PRDM1 as important regulators of PIP3-sensitive mRNAs involved in cell movement.

## INTRODUCTION

Class I phosphoinositide-3-kinases (PI3Ks) are lipid kinases that play key roles in the signal transduction events that allow cell surface receptors to control complex cellular responses, such as growth, movement, proliferation, differentiation and survival (1). Class IA PI3Ks are heterodimers composed of one catalytic subunit (p110α, β or δ) and one of five potential regulatory subunits (p85α/p55α/p50α, p85β or p55γ) (1). Class IB PI3Ks are heterodimers of one catalytic subunit (p110γ) and one of two potential regulatory subunits (p84 or p101) (2). Class IA PI3Ks are adapted to regulation by receptor tyrosine kinases, such as EGF-Rs, PDGF-Rs and insulin/IGF-receptors (3). Class IB PI3Ks are adapted to regulation by GPCRs (2). There is also good evidence that p110β can be be regulated by both GPCRs and RTKs (4).

Upon activation of appropriate cell surface receptors, Class I PI3Ks catalyze the phosphorylation of phosphatidylinositol-4,5-bisphosphate (PI(4,5)P2) to produce phosphatidylinositol-3,4,5-trisphosphate (PIP3) in the plasma membrane (1–3). PIP3 levels are also controlled through its degradation by specific lipid phosphatases; PTEN removes the 3-phosphate to convert PIP3 back into PI(4,5)P2 (5); SHIP1/2 and INPP4A/B sequentially remove the 5-phosphate and 4-phosphate to form PI(3,4)P2 and PI3P, respectively (6,7).

The generation of PIP3 initiates a complex signalling web through the recruitment and regulation of several PH-domain containing proteins at the plasma membrane (1–3,8). These direct effectors include serine/threonine protein kinases (e.g., AKT/PKB), tyrosine kinases (e.g., BTK) and several GEFs and GAPs for small GTPases of the Ras superfamily (e.g., GRP1, ARAP3). These initial effectors are then involved, usually in coordination with other signalling networks, in the regulation of several important cellular responses, such as growth and migration (1–3). One of the best studies pathways is the PKB-dependent regulation of the central regulator of cell growth, mTORC1 (9).

Initial effects of PI3K pathway activation are propagated by allosteric and post-translational effects (e.g., phosphorylation) but there is strong evidence that the pathway exerts important longer term effects through the regulation of translation, mRNA stability and direct regulation of transcription factors, processes in which PKB-mediated phosphorylation is thought to play a critical initiating role (e.g., through PKB-mediated phosphorylation of TSC2, BRF1 or FOXOs) (10–12). Several important homeostatic feedback loops have also been identified (e.g., mTORC1-mediated downregulation of upstream signalling) (13) and there is emerging evidence that expression of critical components of the pathway are regulated by microRNA networks (e.g., mirRNA-mediated regulation of PTEN) (14), all of which act to control the ‘poise’ of the pathway. Some of the proteins involved in PIP3 signalling can also act independently of their effects on the lipid. Indeed it has been described that PTEN localized in the nucleus can also regulate transcription independently of its lipid phosphatase activity via a direct interaction either with the nuclear proteins p53 (15) and MSP58 (16) or with histone H1 keeping the chromatin in a condensed conformation (17). All of these effects make it extremely difficult to track the effects of PI3K pathway activation beyond the first few minutes of receptor stimulation.

The central importance of the PI3K/PIP3 signalling pathway in cell growth is illustrated by the prevalence of driver mutations in this pathway in many different types of human cancer. In breast cancers, oncogenic mutations in the gene encoding the p110α catalytic subunit (PIK3CA) and deletion or truncation of the gene encoding PTEN are present in up to 45 and 40% of human breast tumours respectively, generally in a mutually exclusive manner (18). The most frequent mutations of p110α are found in the kinase domain of the protein (H1047R in exon 20, 47% of the cases) or in the helical domain (E545K or E542K in exon 9, 33% of the cases) and generate an activated kinase (3,19,20). In all cases, PIK3CA and PTEN oncogenic modifications are consistent with the logic that more PIP3 supports more cell growth.

We have attempted to unravel some of the longer term effects of PI3K/PIP3/PTEN signalling on cell function by investigating the effects of both chronic and short term manipulation of this pathway on genome-wide mRNA accumulation in human breast epithelial MCF10a cells. We investigated isogenic cell lines expressing a single allele of H1047R-PIK3CA or loss of both alleles of PTEN to model long term activation of the pathway. We also investigated stimulation with EGF, in the presence or absence of the selective p110α inhibitor A66 (21), to model short term inhibition in the context of either basal or growth factor conditions. Chronic activation of the pathway produced remarkably wide and diverse effects on the mRNA landscape, with much smaller effects of short-term pharmacological inhibition. Analogous large scale-remodelling of the transcriptome and proteome of H1047R-PIK3CA cells has been reported recently (22). A small proportion of changes in mRNA accumulation aligned with the directional logic of the genetic and pharmacological manipulation employed, allowing predictions of mRNAs that may be more directly regulated by the pathway. Further, an analysis of transcription factor binding sites enriched within differentially regulated gene sets allowed the prediction of transcription factors that may play a significant role downstream of PI3K activation and also the components of a novel feedback loop.

## MATERIALS AND METHODS

### Cell lines and tissue culture

MCF10a are non-transformed human breast epithelial cells. PTEN KO (biallelic deletion of exon 2) and PIK3CA H1047R (p110α H1047R, modification of one allele only) MCF10a cell lines generated by targeted homologous recombination were obtained from Horizon Discovery together with their wild-type (WT) parental cell lines. Cells were maintained at 37°C with 5% CO_2_ in DMEM/F12 supplemented with 5% horse serum, 10 ng/ml EGF (except for PIK3CA H1047R cells where no EGF is added), 10 μg/ml insulin, 0.1 μg/ml cholera toxin, 0.2 μg/ml hydrocortisone. The assay medium used for the starvation and RNA-seq assays described below was made of DMEM/F12 supplemented with 1% charcoal dextran treated foetal bovine serum (FBS), 0.1 μg/ml cholera toxin, 0.2 μg/ml hydrocortisone. DMEM/F12 and the charcoal dextran treated FBS were from Life Technologies, the horse serum was from PAA. All the other reagents were from Sigma Aldrich.

### RNA-seq libraries preparation and sequencing

**Figure 1.**
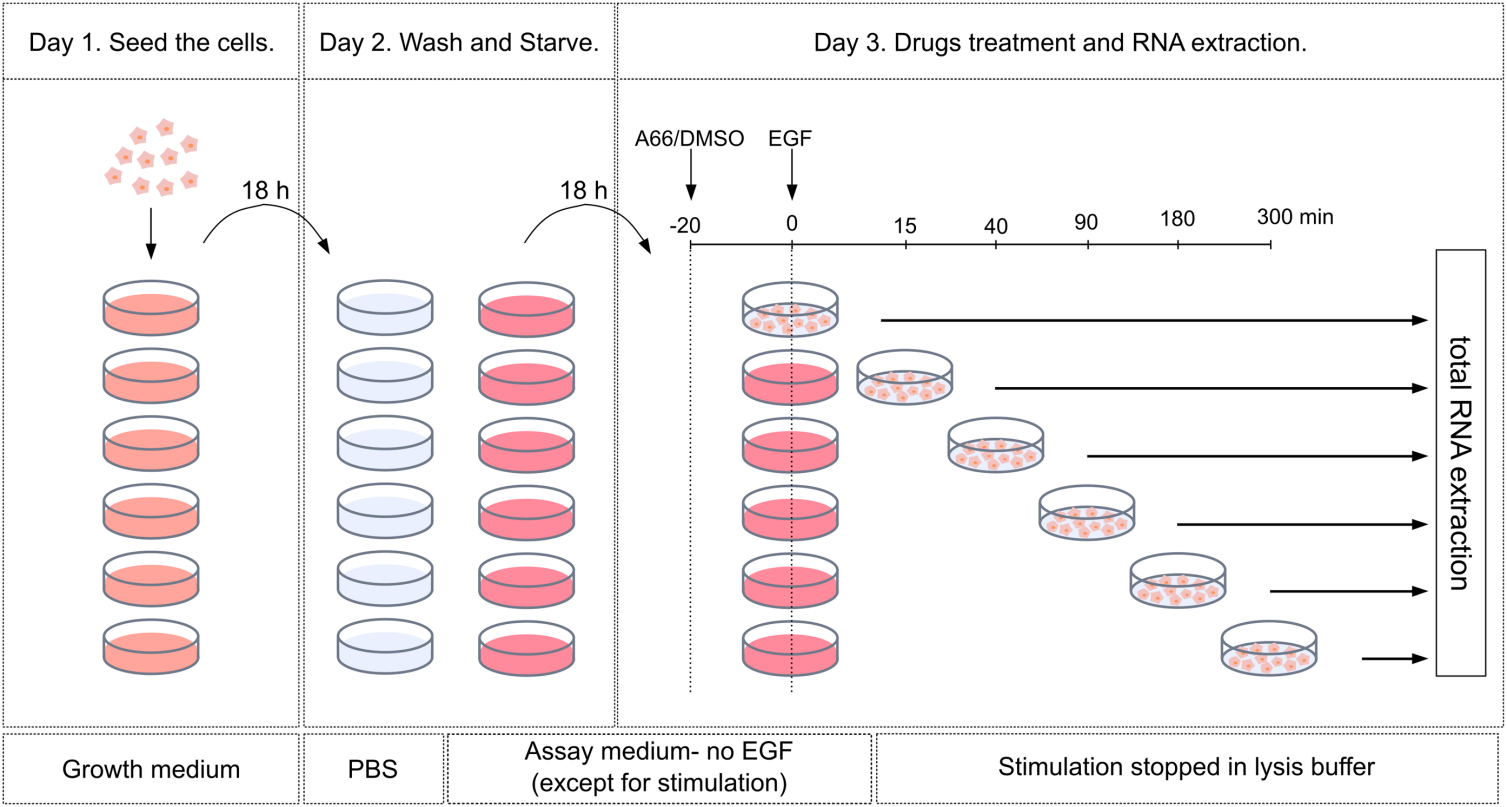
Experimental procedure. Cells were plated in growth medium on day 1, washed and starved (Serum, EGF, Insulin deprivation) for 18 h on day 2. On day 3, cells have been incubated with the p110α inhibitor A66 (2 uM) or DMSO (A66 vehicle) for 20 min prior EGF stimulation (10 ng/ml) for 15, 40, 90, 180 and 300 min or left unstimulated in presence of A66 for 300 min (A66 no EGF samples) then total RNA extraction has been performed.

On day 1, cells were seeded at 320 000 cells in 60 mm dish in their growth medium (see above) and left to adhere at 37°C for 18 h (Figure 1). On day 2, the growth medium is replaced with the assay medium (see above) after the cells were washed with phosphate buffered saline. Cells are starved in serum, EGF and insulin for 18 h at 37°C. On day 3, cells were pre-incubated with either A66 (p110α specific inhibitor http://identifiers.org/pubchem.compound/42636535, obtained from Selleckchem, 2 μM) or DMSO only (vehicle for A66) for 20 min, then incubated with EGF (10 ng/ml) +/− A66/DMSO or DMSO for a further 15, 40, 90, 180 or 300 min. The reactions were stopped by carefully discarding the medium and adding the RLT lysis buffer provided in the RNeasy kit (from QIAGEN). Total RNA extraction was performed as recommended by the manufacturer. The integrity of the total RNA was controlled on a 1% agarose gel (not shown). RNA-seq libraries were prepared from 4 μg total RNA using the Truseq RNA sample preparation kit (from Illumina). Amplified libraries were assessed for quality and quantity using High-Sensitivity DNA chips on the Agilent Bioanalyzer and the KAPA Library Quantification Kit for Illumina (KAPA Biosystems). Pools of six libraries were prepared for 100-bp single-end sequencing on a HiSeq 2500. The dataset supporting the results of this article is available in the Gene Of Expression (GEO) repository, accession number GSE69822.

### RNA-Seq data processing and differential expression analysis

Quality control of raw fastq files obtained from the sequencing machine was performed using FastQC (23). All 75 samples were checked for sequencing quality and in all of them three main quality checks (Basic Statistics, Per base sequence quality and Per sequence quality scores) were passed. Reads were mapped to a human reference genome (Ensembl GRCh37 release 75) by TopHat splice junction mapper for RNA-Seq (24). About 85–95% of reads in every sample were uniquely mapped, with the majority of samples having >90% uniquely mapped reads. The number of reads per gene was counted with HTSeq (25) and a read-count matrix was constructed. DESeq2 Bioconductor package (26) was used to perform differential gene expression analysis. Three types of comparisons were used: (i) Pairwise comparison between two time points from two different conditions (the design formula in the generalized linear model (GLM) contains only one condition/time factor); (ii) comparison over multiple time points (time course) of gene expression in a single condition (the design formula in the GLM contains a time factor; then the likelihood ratio test is used with the reduced model where the time factor is removed from the design formula); (iii) comparison of two time courses from two different conditions (the design formula in the GLM contains a condition factor, a time factor and an interaction of the two; then the likelihood ratio test is used to test the significance of both condition differences and condition-time interaction with a reduced model which does not contain both the condition and the interaction terms). 0.05 significance level of adjusted *p*-values was used in all pairwise comparisons. 0.01 significance level of adjusted *p*-values was used in time course comparisons. For plotting time courses of RNA-Seq data, samples were normalized by Size Factors obtained by ‘estimateSizeFactors’ function from DESeq2. When the expression of different genes needed to be compared (e.g. Table 2), it was normalized as reads per kilobase per million reads (RPKM). Expressions of each gene in all conditions (with either only Size Factor normalization or with RPKM values) can be accessed at: http://www.bioinformatics.babraham.ac.uk/shiny/kiselev-pip3-rna-seq-gene-profiles/.

**Table 1.** Results of ISMARA analysis (see Materials and Methods) for the most strongly regulated (S_*m*_ > 20) genes from gene sets 1–4 and A-H

TF binding motif	Motif target gene	Sm	Gene set
PRDM1	ATF3	79.5	1, C
PRDM1	KCNB1	28.0	3, A
PRDM1	APOL3	25.5	1, C
PRDM1	NCOA7	23.8	1, A
PRDM1	TLR3	22.3	1, G
PRDM1	LIF	20.2	1, A
SRF	EGR1	78.3	1, A
SRF	EGR2	61.5	1, A
SRF	NR4A1	48.8	1, C
SRF	DUSP5	40.7	1, A
SRF	EGR4	37.2	1, E
SRF	DUSP2	32.5	1, E
SRF	EGR3	31.8	1, E
SRF	FOSL1	26.0	1, E
SRF	FOSB	20.2	1, G
TBP	KRTDAP	51.9	D
TBP	KRT1	43.1	D
RREB1	NGFRAP1	51.2	1, A
RREB1	PCSK1N	35.9	4, E
RREB1	FEZ1	28.1	B
RREB1	COL7A1	22.0	1, E
GTF2I	KLK7	47.8	1, A
GTF2I	BGN	30.8	D
GTF2I	EGR3	27.3	1, E
EHF	MAGEB2	46.7	F
EHF	ADPRHL1	40.6	E
EHF	TMEM156	25.3	B
HBP1_HMGB_SSRP1_UBTF	SOX6	46.2	G
HBP1_HMGB_SSRP1_UBTF	FBXO32	45.3	1, A
HBP1_HMGB_SSRP1_UBTF	FOSB	38.0	1, G
HBP1_HMGB_SSRP1_UBTF	ANGPT1	30.1	1, A
HBP1_HMGB_SSRP1_UBTF	FOXL1	27.8	1, C
HBP1_HMGB_SSRP1_UBTF	PNRC1	26.9	A
HBP1_HMGB_SSRP1_UBTF	SEMA6D	25.0	1, A
HBP1_HMGB_SSRP1_UBTF	VAV3	22.0	3, C
HBP1_HMGB_SSRP1_UBTF	SMAD6	20.8	E
ZNF143	ZNF223	42.8	H
ZNF143	ZNF527	21.0	E
ZNF143	ZNF331	20.1	E
YY1	KRT13	36.6	2, B
YY1	FTL	22.4	A
YY1	ONECUT3	20.5	E
ATF5_CREB3	MAP1LC3A	34.1	B
ATF5_CREB3	NR4A1	26.2	1, C
TFDP1	FST	32.7	E
TFDP1	PTHLH	20.4	1, E
NRF1	PDZRN3	27.8	F
NRF1	MYPOP	25.6	F
GATA6	KLK10	27.0	1, C
E2F1..5	E2F2	26.5	H
E2F1..5	SNRPN	24.1	F
E2F1..5	SNURF	24.1	H
E2F1..5	MYB	23.4	E
GATA1..3	ATF3	26.1	1, C
TGIF1	KRT13	26.0	2, B
NFKB1_REL_RELA	GPAT2	23.6	B
NFKB1_REL_RELA	NR4A1	22.5	1, C
NFKB1_REL_RELA	CFB	20.0	2, D
SP1	PTPRN2	22.8	E
bHLH_family	EIF5A2	21.7	1, G
ARNT_ARNT2_BHLHB2_MAX_MYC_USF1	RAB3IL1	28.4	G
ARNT_ARNT2_BHLHB2_MAX_MYC_USF1	EIF5A2	21.2	1, G
ARNT_ARNT2_BHLHB2_MAX_MYC_USF1	AKAP12	21.0	1, A
KLF4	DNER	20.2	A
KLF4	WT1	20.1	A
TFAP2B	RBPMS2	20.1	E

**Table 2.** List of genes from set A regulated by PRDM1 transcription factor binding motif

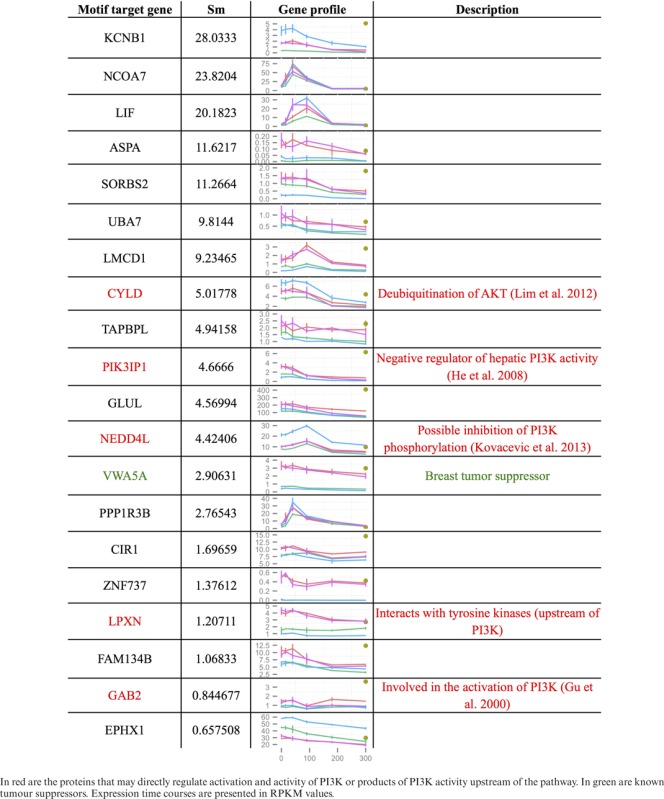			

### Correlation and principal component analysis (PCA) analysis

Correlation coefficients between samples were calculated as Pearson's correlation coefficients using the R function *cor*. PCA was performed by using the R function *prcomp*.

### Gene clustering

Partitioning around medoids (*k*-medoids) (27) was used to cluster genes in identified gene sets. R base *pam* function was applied to scaled and centred subsets of the read-count matrix. The number of clusters, *k*, was varied in the interval from 2 to *k*_max_, where *k*_max_ was uniquely defined for each gene set (depending on the number of genes). To assess a clusterwise stability and find an optimal number of clusters for each gene set we used a bootstrapping strategy where Jaccard similarities were calculated for each resample of each cluster (R package *fpc* and (28,29)). Hundred resamples of each cluster were performed for each *k*. Jaccard similarities from all resamples were then plotted (Supplementary Figures S1 and S2). We defined a cluster as stable if its median Jaccard similarity value was ≥0.75 and its standard deviation was ≤10% of the mean. Jaccard similarities are generally high for a small number of clusters (2–4). They tend to become lower for intermediate number of clusters and they recover back to a high level (>0.75) when number of clusters is large. In general, we define an optimal k for a given gene set as the maximum *k* (in a small or intermediate range of *k*s) at which all clusters are still stable.

### Gene ontology (GO) analysis

Gene ontology (GO) analysis was performed using classical Fisher test with topGO Bioconductor package (30). A *p*-value of 0.05 was used as the level of significance. Significant GO terms were then summarized by removing redundant GO terms using REVIGO (31). On the figures only GO terms with *p*-value < 1e-4 are shown. The WebGestalt web tool (32) was also used to confirm the results of topGO package.

### ISMARA analysis

To analyse activities of transcription factor binding motifs (TFBM) using RNA-Seq data, we used the Integrated System for Motif Activity Response Analysis (ISMARA) (33). The results of ISMARA analysis can be accessed using this link: http://lenoverelab.org/data/2015/kiselev/ismara_report_hg19/. One of the ISMARA outputs is the S_*pm*_ score, which shows how much the deviation between the predicted and observed signal in the statistical model would increase if the motifs *m* were removed from the promoter *p*. In other words, S_*pm*_ shows the strength of the regulation by motif *m* of the target gene controlled by the promoter *p*. In our analysis, we only used promoters that have RefSeq identifiers. When a target was present several times with different promoters, we used the promoter which had the maximum score S_*pm*_ (personal communication with Piotr Balwierz). This allowed us to have a unique score S_*pm*_ per target for every motif. Insignificant motifs with *z*-value < 2 were not considered. To compare two distributions of S_*m*_ (scores for a given motif) we used two non-parametric tests: Kolmogorov–Smirnov test (R function *ks.test*) and Mann–Whitney test (R function *wilcox.test*). See Supplementary Tables S1 and S2.

### R package

An R package containing all datasets described in the paper and scripts/functions needed to reproduce them, can be installed from: https://github.com/wikiselev/rnaseq.mcf10a.

## RESULTS

This study aimed at defining the role of PIP3 signalling in EGF-induced regulation of mRNA in the non-transformed human breast epithelial cells MCF10a, as an indication of its involvement in transcription regulation and/or mRNA stability. Genome-wide mRNA profiling was performed by RNA-Seq on WT MCF10a cells starved overnight then stimulated with 10 ng/ml EGF for 15, 40, 90, 180 and 300 min (Figure 1). Those libraries and resulting data are called ‘WT’ in the rest of the article. Libraries were produced using the Truseq RNA sample preparation kit from Illumina as described in the Materials and Methods. All the RNA-Seq data has been deposited in the Gene Expression Omnibus under the accession number GSE69822.

Previous work with these cells showed that PI3Kα is the main PI3K class I isoform activated upon EGF stimulation (34). Thus WT cells were pre-treated with the p110α-selective inhibitor A66 (2 μM) or DMSO for 20 min after the starvation period and before EGF stimulation, as well as during EGF stimulation (same time points as described above; referred to as ‘A66’). In addition WT cells were left with A66 without EGF for a further 300 min (referred to as ‘A66 no EGF’) to inhibit PI3Kα basal activity.

PI3K pathway is frequently disrupted in breast cancer; in particular the p110α subunit is often mutated at specific hot spots whereas PTEN expression is often abolished, all leading to a constitutive activation of the PI3K pathway. In order to analyse the effect of these individual perturbations in both the presence and absence of EGF we used isogenic MCF10a cell lines either expressing a constitutively active version of p110α (referred to as ‘PIK3CA H1047R’, these cells have one WT and one mutated allele) or deleted for both PTEN alleles (referred to as ‘PTEN KO’). The presence of the H1047R mutation in the PIK3CA H1047R cells was confirmed by sequencing and the absence of PTEN in the PTEN KO cells by western-blot (data not shown). Both cell lines were submitted to the same starvation/EGF stimulation protocol as the WT MCF10a cells.

All libraries were sequenced in triplicates using the HiSeq 2500 (100 bp single-end reads, the average number of reads in a library is 31 million). The samples analysed can be summarized as,

**-WT**: WT MCF10a unstimulated or stimulated by EGF for 15, 40, 90, 180, 300 min in the presence of DMSO (pre-incubation with DMSO for 20 min before EGF stimulation).
**-A66**: WT MCF10a unstimulated or stimulated by EGF for 15, 40, 90, 180, 300 min in the presence of A66 (pre-incubation with A66 for 20 min before EGF stimulation).
**-A66 no EGF**: WT MCF10a incubated with A66 alone for 20 min pre-incubation plus an additional 300 min.
**-PTEN KO**: PTEN knockout MCF10a unstimulated or stimulated by EGF for 15, 40, 90, 180, 300 min in the presence of DMSO (pre-incubation with DMSO for 20 min before EGF stimulation).
**-PIK3CA H1047R**: p110α ^H1047R/+^ knock-in MCF10a unstimulated or stimulated by EGF for 15, 40, 90, 180, 300 min in the presence of DMSO (pre-incubation with DMSO for 20 min before EGF stimulation).

### Correlation and principal component analysis of the mRNA-seq data

**Figure 2.**
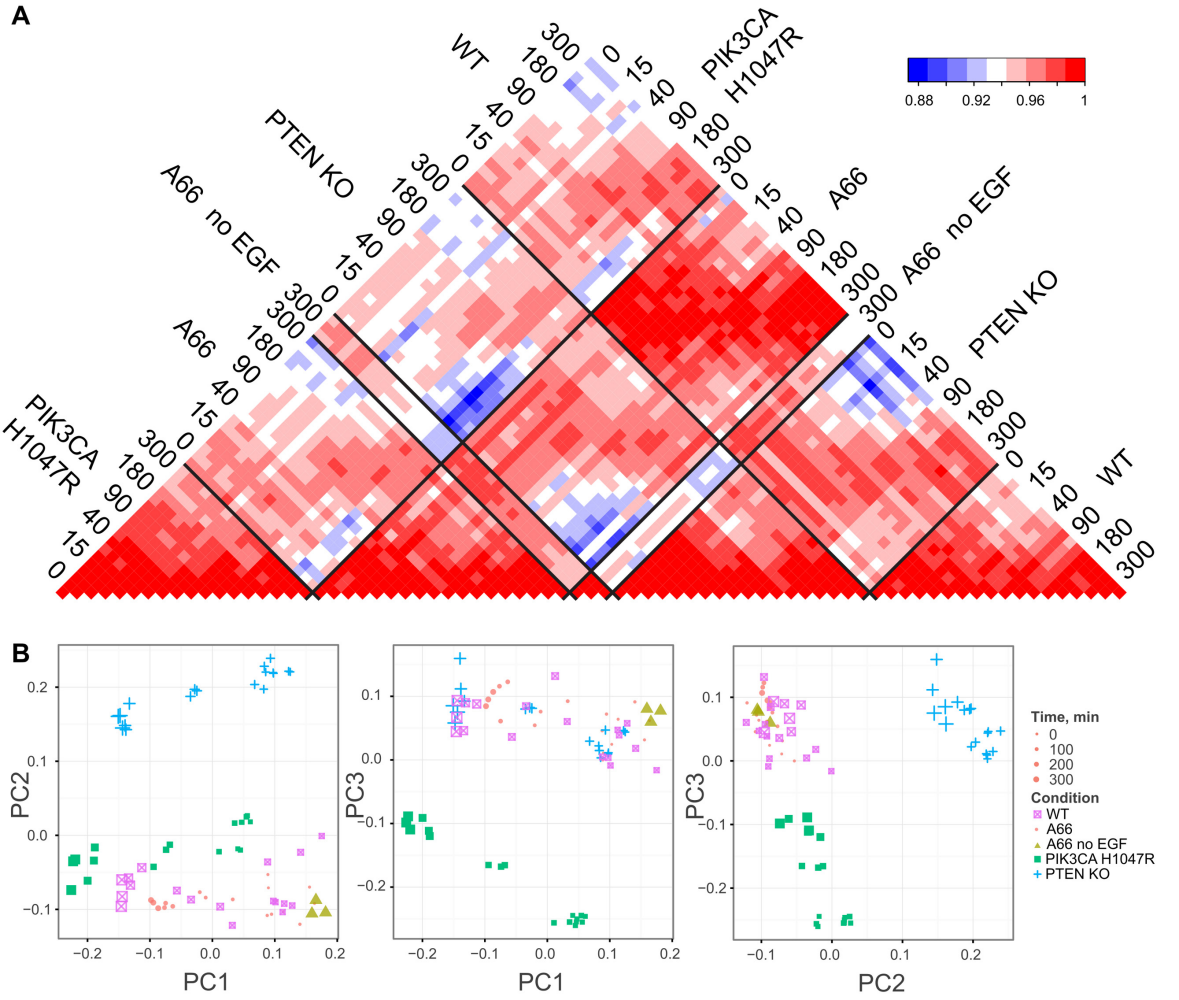
(**A**) Correlation matrix of all 75 RNA-Seq samples (based on Pearson correlation coefficients). Each labelled row contains three technical replicates; (**B**) Results of the principal component analysis performed on all 75 RNA-Seq samples. Smaller size of elements corresponds to early time points (0–40 min), large size of elements corresponds to later time points (180–300 min).

Both correlation analysis and principal component analysis (PCA) of all 75 RNA-Seq samples (Figure 2A and B) showed that experimental triplicates were highly correlated, with no strong outliers, and showed very little biological variation. The lowest correlation coefficients were around 0.8, reflecting the fact that most genes are not affected by the perturbations. In addition, PCA also shows that the main source of variance in the data (represented by principal component 1 — PC1) corresponds to changes due to the duration of EGF stimulation (sizes of the symbols in Figure 2B). PC2 and PC3 represent the variance in the data introduced by PTEN KO and PIK3CA H1047R, respectively. In agreement with the correlation analysis (Figure 2A) and the experimental design, WT and A66 samples are the closest in the principal component space and indicate that over these time scales A66 treatment does not significantly alter the WT cell line. Conversely, PTEN KO and PIK3CA H1047R are largely separated from WT/A66 and from each other which reflects the basal effects of mutations on the cell lines. Taken together, this showed that the treatment with the p110α-selective inhibitor A66 had a significant but limited effect on the changes of mRNA levels induced by EGF in WT MCF10a cells, suggesting that the EGF response is largely mediated, as expected, by PI3K independent pathways (e.g., MAP kinase pathways). This also indicates that the presence of the point mutation PIK3CA H1047R or the deletion of PTEN in MCF10a induced long term changes in the transcriptional landscape, generating cell types different from the WT cell line and from each other. Thus in the rest of the work, we separated the analysis of the WT cell line from the analysis of isogenic mutant cell lines.

### PI3K-dependent regulation of mRNA levels induced by EGF in the WT

**Figure 3.**
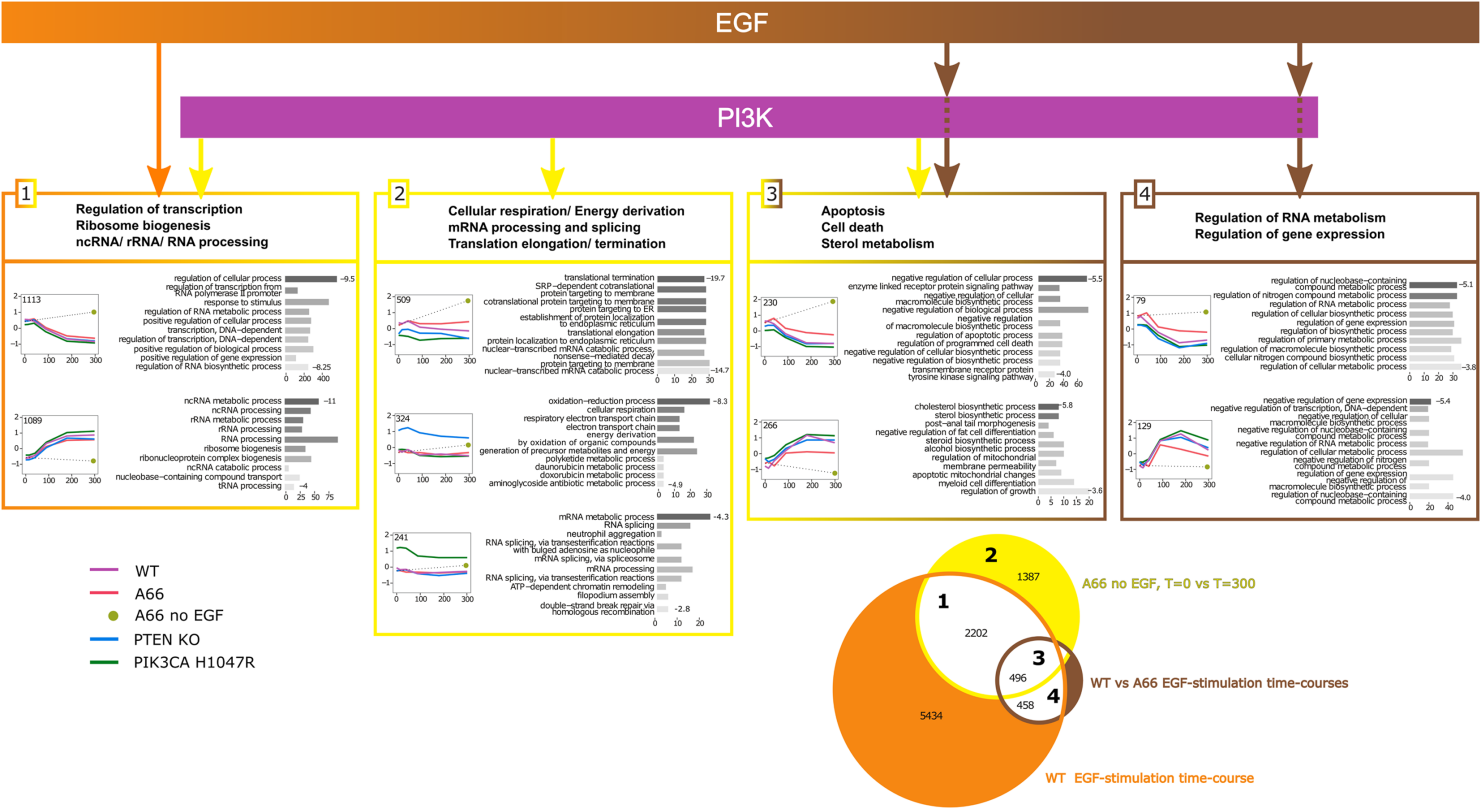
Summary diagram of PI3K mediated functions based on the analysis of WT cell lines. Arrow colours correspond to colours of the Venn diagram in the bottom-right corner of the figure. The Venn diagram represents an overlap of three gene sets identified by differential expression analysis (see text for description). Gene expression time course profiles represent the average of many genes (exact number is shown in each profile). Only the gene clusters with significant GO enrichment are shown. Values on the right of GO bar charts are log10(*P*-value) for the corresponding terms. Grey levels reflect the significance. Bar lengths represent the number of genes annotated by the GO term.

To analyse the WT cell line we compared its mRNA landscapes (see Materials and Methods): (i) between 300 and 0 min in A66 no EGF condition and 2) between time courses of A66 and WT in the presence of EGF. The resulting two mRNA sets represent mRNAs affected by the *basal activity of PI3K* and the *EGF-induced activity of PI3K*, respectively. These mRNA sets were further overlapped with all mRNAs responding to EGF stimulation in the WT (differentially expressed between any of the time points of EGF time course in WT and referred to as EGF-dependent in WT in the rest of the paper); see Venn diagram in Figure 3. The overlap created 7 new mRNA sets. GO enrichment analysis of these sets provided only very general and not significantly enriched GO terms. In order to identify the main biological functions of the genes in each set, we clustered them according to their time course profiles (Supplementary Figure S1) and performed GO enrichment analysis for each resulting cluster (Figure 3). We further considered sets 1, 2, 3 and 4 (other sets are either not related to PI3K pathway, can not be properly clustered or their clusters are not significantly enriched with GO terms).

#### mRNAs affected by the basal activity of PI3K

The expression of 1387 mRNAs in set 2 was only affected by the *basal activity of PI3K* (yellow arrows in Figure 3). EGF treatment has no effects on these mRNAs. They can be arranged in four stable clusters. In three of these clusters, the genes encoding the mRNAs are significantly enriched for GO terms. The genes encoding the mRNAs that were generally overexpressed in PTEN KO are involved in cellular respiration and energy metabolism. In contrast, the genes encoding the mRNAs that were generally overexpressed in PIK3CA H1047R are involved in mRNA processing and RNA splicing. The cluster showing the most significant enrichment (*p*-value ≈ 1e-19) contains genes involved in translation elongation/termination: these mRNAs were generally slightly lower in both PTEN KO and PIK3CA H1047R cell lines while strongly increased in the presence of the A66 no EGF condition.

#### mRNAs affected by both basal activity of PI3K and other EGF-dependent pathways

The expression of 2202 mRNAs in set 1 was affected by both the *basal activity of PI3K* described above and by other EGF-induced pathways independent of PI3K (combination of orange and yellow arrows in Figure 3). These mRNAs were clustered in two large stable clusters containing over 1000 mRNAs each. One cluster contains genes encoding mRNAs whose levels peaked at early time points of EGF stimulation (15 and 40 min), then steadily declined over the remaining time course; these genes are involved in the regulation of transcription and RNA metabolism. In contrast, the other cluster contains genes encoding mRNAs that reached a peak at later time points (180 and 300 min) and are involved in post-transcriptional processing of non-coding RNA. Interestingly, genes in neither cluster were differentially expressed in PTEN KO nor PIK3CA H1047R.

#### mRNAs affected by EGF-induced activity of PI3K

The levels of 458 mRNAs in set 4 were only affected by the *EGF-induced activity of PI3K* (brown arrow in Figure 3). The mRNAs could be clustered into four stable clusters. The genes encoding the mRNAs of two of these clusters were significantly enriched with GO terms; these are involved in the regulation of RNA metabolism and regulation of gene expression. Interestingly, the genes encoding the mRNAs whose levels peaked at later times (>90 min) are more specifically implicated in a negative regulation of those processes.

#### mRNAs affected by both basal and EGF-induced activities of PI3K

The levels of 496 mRNAs in set 3 were affected by both *basal* and *EGF-induced activities of PI3K*. The mRNAs were clustered in two stable groups. Genes encoding the mRNAs whose levels were relatively high at early time points (15 and 40 min) and then reduced by EGF treatment are involved in apoptosis and cell death. Genes encoding the mRNAs that were increased by EGF treatment are mainly linked to sterol metabolism, although apoptosis appeared as well.

### General overview of transcriptional regulation based on ISMARA analysis

**Figure 4.**
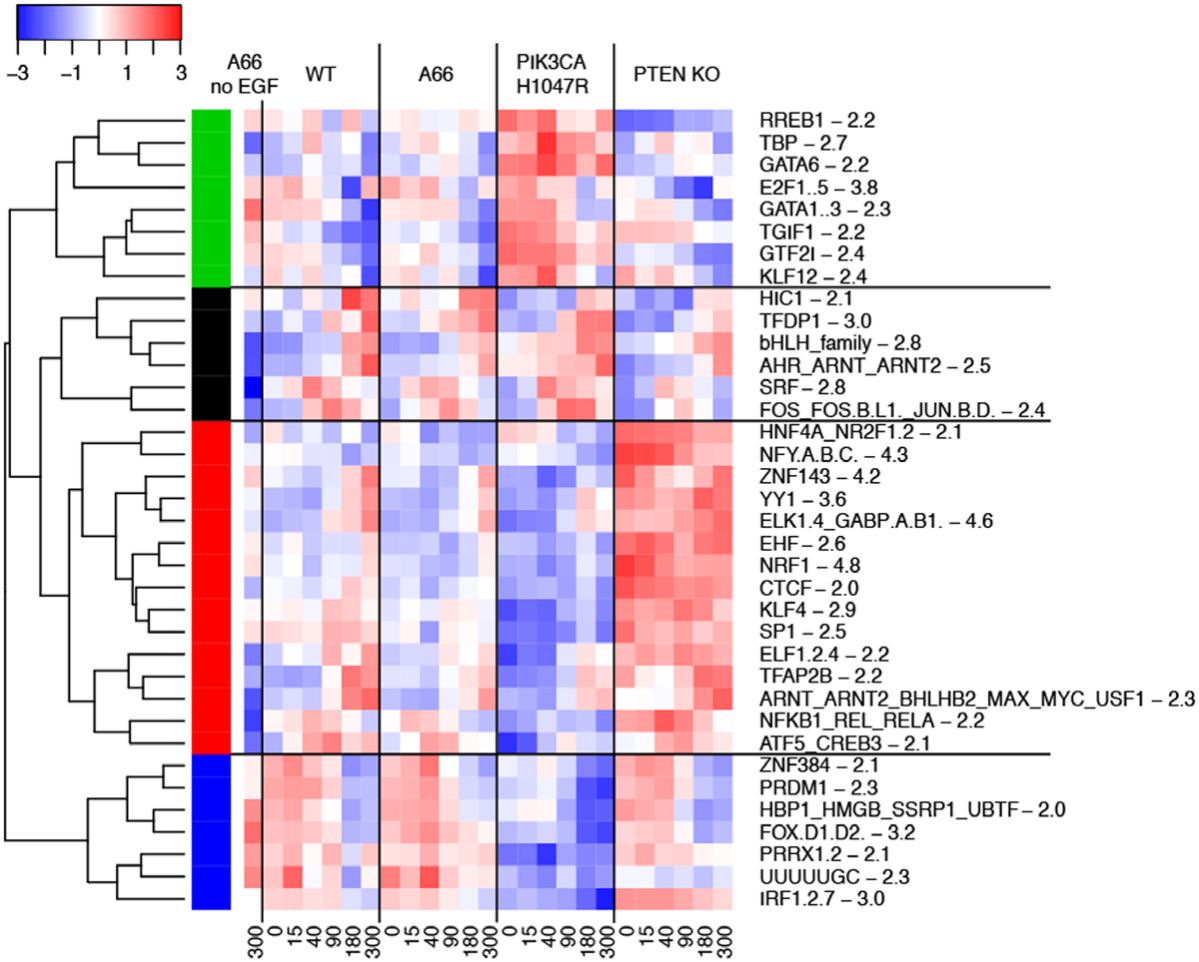
A heatmap of averaged (scaled and centered) activities of TFBMs from the ISMARA analysis. Different colours on the dendrogram correspond to different clusters of TFBMs. The values next to the TFBM names represent ISMARA's *z*-values.

To identify how PIP3 signals might control mRNA levels we performed an analysis of the RNA-Seq data using ISMARA (see Materials and Methods). The main output from such an analysis is the activity of transcription factor binding motifs (TFBMs) in every sample. A summarized heat map of activities of the most significant (*z*-value > 2) TFBMs is shown in Figure 4. These activities resolve into four clusters: (i) where motifs’ activities are increased in PIK3CA H1047R cells and decreased by EGF (green cluster); (ii) where motifs’ activities are increased by EGF but not modified by PIP3 perturbations (black cluster); (iii) where motifs’ activities are increased in PTEN, decreased in PIK3CA H1047R and increased by EGF (red cluster); (iv) where motifs’ activities are decreased in PIK3CA H1047R and by EGF treatments (blue cluster). Interestingly, all the most significant motifs (*z*-value > 4) belong to the red cluster 3, suggesting that PTEN KO has the strongest effect on the cells. Since motif activities in ISMARA are calculated using RNA-Seq data we found similar trends of behaviour in actual gene expression profiles (see Discussion about mutant cell lines). However, it is important to note that some TFBMs bind repressors, therefore an increase in their activity would tend to decrease gene expression.

### Transcriptional regulation of genes affected by PI3K in WT cell lines

ISMARA also provides the strengths of gene regulation by different TFBMs, i.e. for each TFBM *m* and any of its target genes ISMARA computes the overall strength of regulation of the target by the TFBM (S_*m*_ score). To better understand how mRNAs in sets 1–4 were transcriptionally regulated we looked at the most strongly regulated genes (S_*m*_ > 20) in these gene sets (Table 1). The most highly regulated genes belong to set 1. The six main regulating motifs (ISMARA nomenclature) are PRDM1, SRF, RREB1, GTF2I, EHF and HBP1_HMGB_SSRP1_UBTF. The SRF motif (representing the Serum Response Element (SRE), binding the transcription factor SRF) strongly affected EGRs, NR4A1, DUSP2, DUSP5, FOSL1 and FOSB, genes that are all known to be regulated by the ERK pathway. This result confirms the cross-talk between PI3K and other EGF-dependent signalling pathways. PRDM1 (representing the PRDM1 element, that binds the transcription factor BLIMP1) was another interesting motif that strongly regulated ATF3 and some other genes. Other transcription factors known to be regulated through the PI3K pathway (e.g. FOXO) were also retrieved from the ISMARA analysis but with lower scores.

### PI3K-dependent regulation of mRNA levels in PTEN KO and PIK3CA H1047R cells

**Figure 5.**
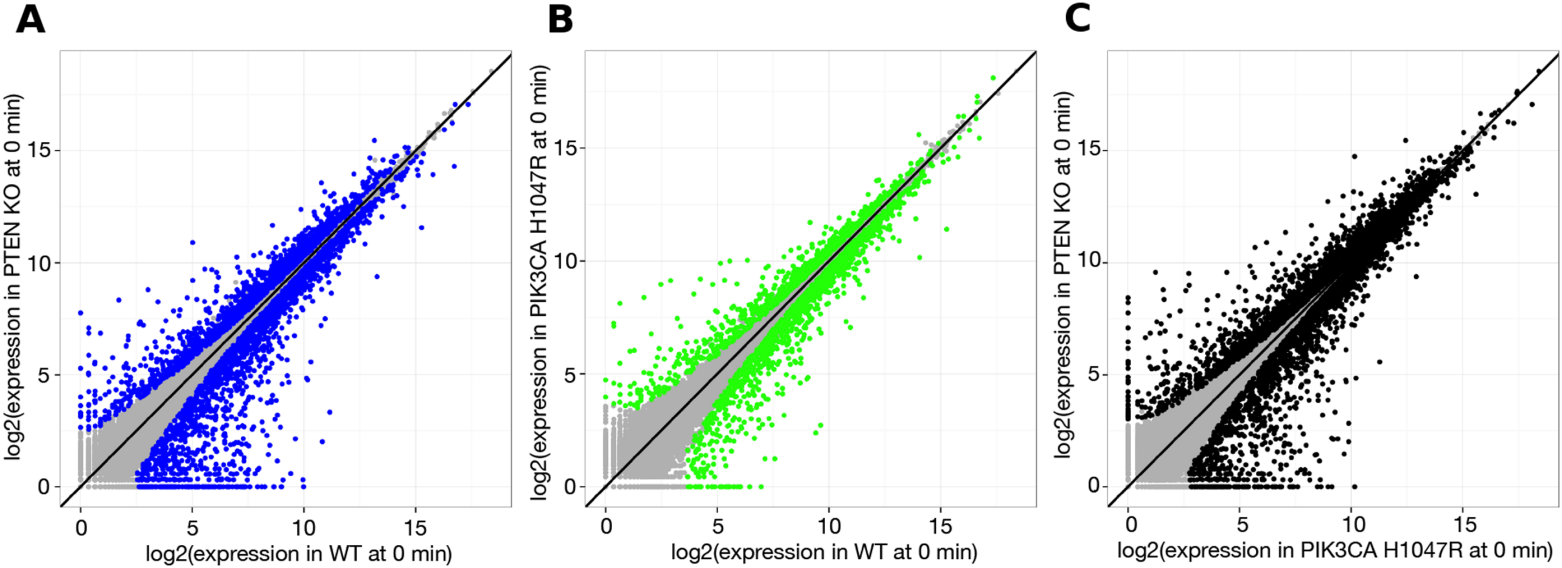
Scatterplots of mRNA levels comparing WT, PTEN KO and PIK3CA H1047R conditions at 0 min (in the absence of EGF). mRNAs which levels are significantly different are coloured with blue (**A**: WT versus PTEN KO), green (**B**: WT versus PIK3CA H1047R) and black (**C**: PTEN KO versus PIK3CA H1047R).

To analyse the mutant cell lines (PTEN KO and PIK3CA H1047R), we compared their mRNA landscapes (see Materials and Methods):

- Between genetically modified and WT cell lines in the absence of EGF (at 0 min). A total of 3628 and 2345 mRNAs were significantly changed by loss of PTEN or expression of p110α-H1047R respectively. Scatterplots of mRNA levels under these two conditions at 0 min are shown in Figure 5. These gene sets represent the *chronic* effects of the genetic modifications;
- Between time courses of genetically modified and WT cell lines in the presence of EGF. A total of 7825 and 7486 mRNAs were identified in the PTEN-KO and p110α-H1047R-expressing cell lines respectively. These mRNA sets represent changes in the *EGF-induced* effects by the genetic modifications. They were combined together resulting in a set of 11000 mRNAs, the expression of which was affected by the presence of either or both mutations.

**Figure 6.**
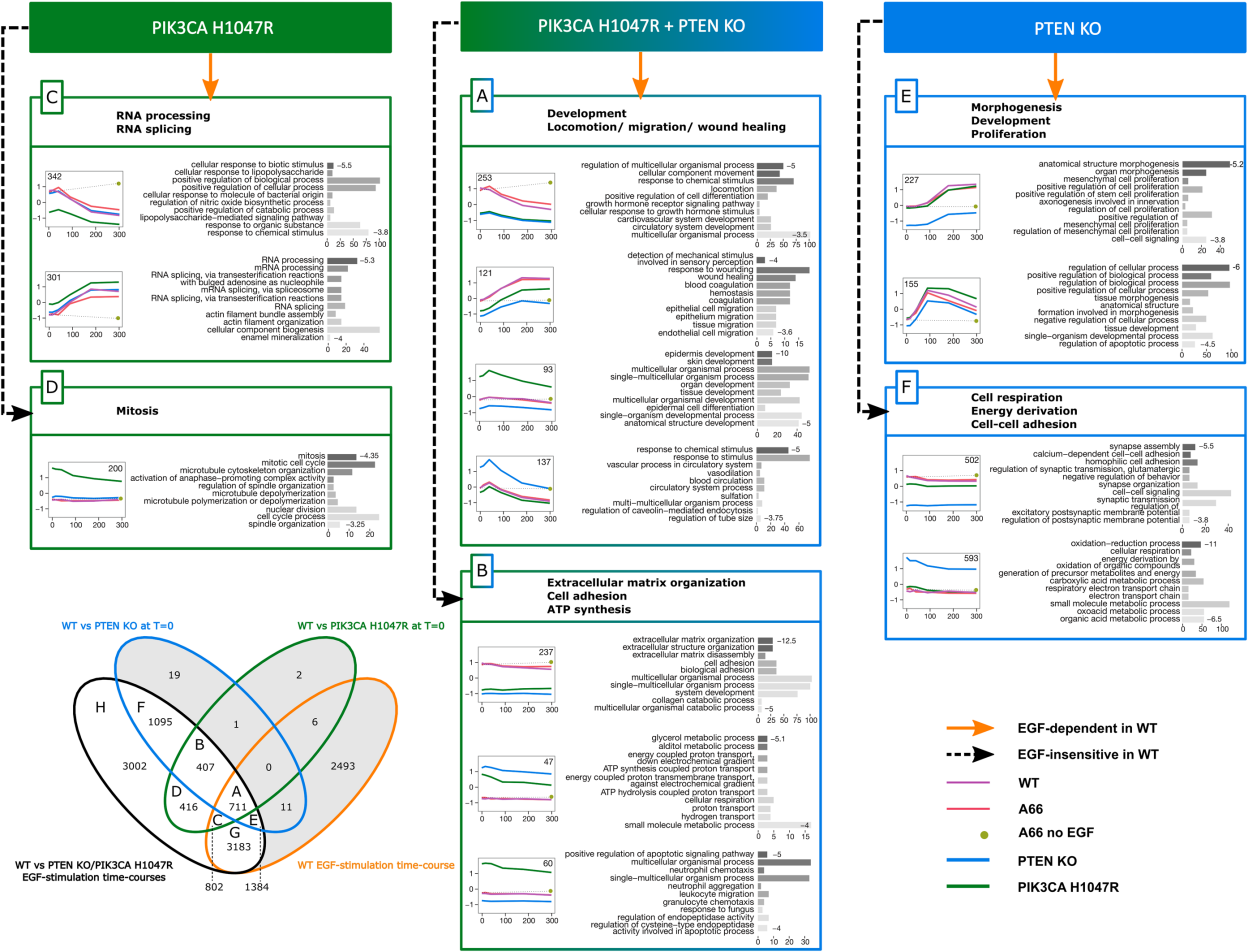
Concise diagram of activities of genes with constitutive mutational effects. Box colours correspond to colours of the Venn diagram in the bottom-left corner of the figure. Venn diagram represents an overlap of three gene sets identified by differential expression analysis (see text for description). Gene expression time course profiles represent an average of several genes (exact number is shown in each profile). Values on the right of gene ontology bar charts are log10(*P*-value) of corresponding ontology terms. Concise description of each gene set (**A**–**F**) is based on its ontology terms and is shown in bold font at the top of every gene set box.

The overlap between sets of mRNAs corresponding to *chronic* and *EGF-induced* effects of the mutations and all EGF-dependent mRNAs in WT (Venn diagram in Figure 6) created 15 new mRNA sets. We further analysed sets A to H (white background on the Venn diagram) using the procedure described above for the WT cell lines. We clustered them according to their time course profiles (Supplementary Figure S2) and performed a GO enrichment analysis for each resulting cluster (Figure 6). Note that mRNA sets identified in the analysis of WT cell lines and in the analysis of mutant cell lines may overlap.

#### EGF-induced effect of the mutations

**Figure 7.**
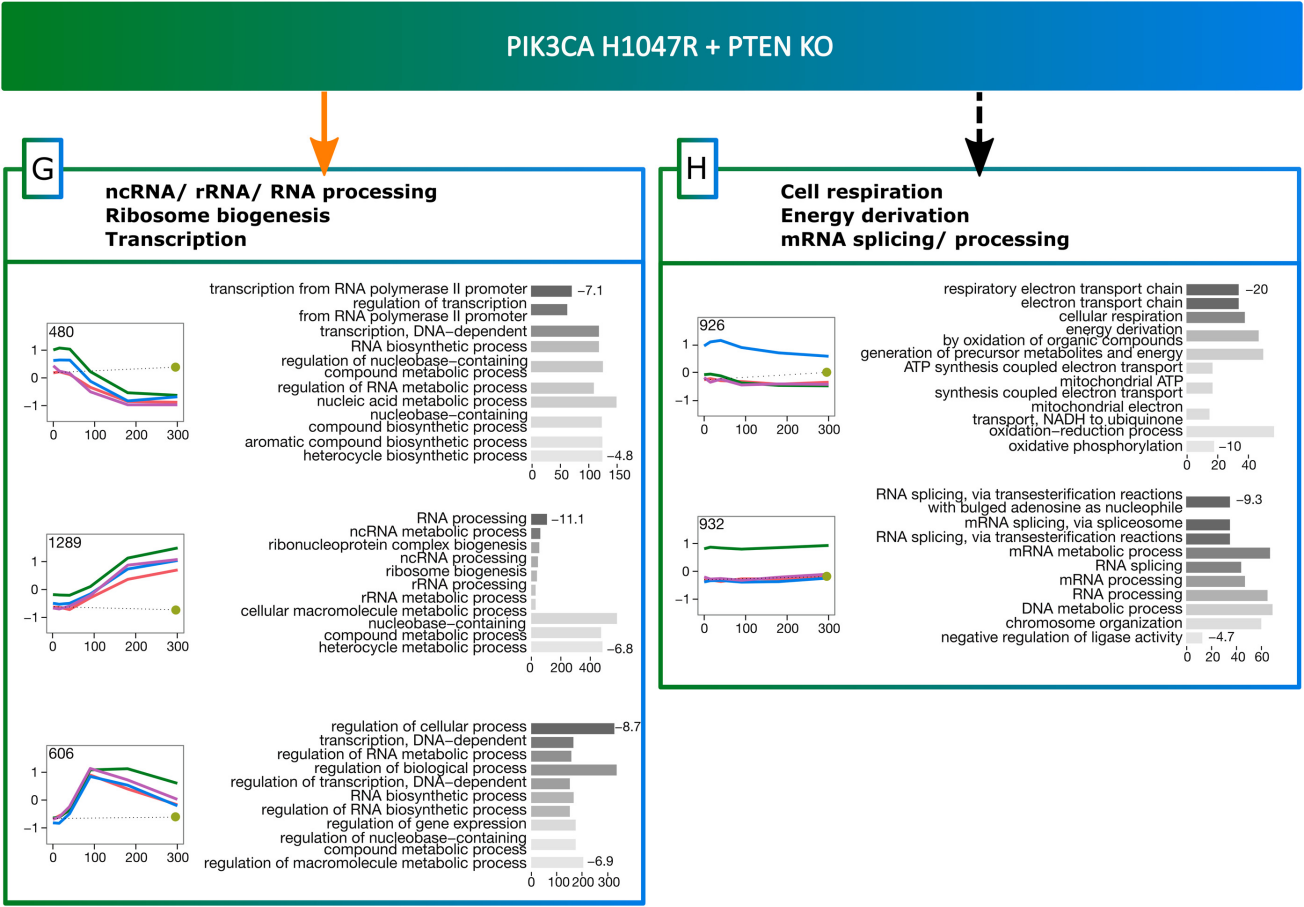
Concise diagram of activities of genes with induced mutational effects. Box colours correspond to colours of the Venn diagram in the bottom-left corner of Figure 5. Gene expression time course profiles represent an average of several genes (exact number is shown in each profile). Colour legend of gene expression time course profiles is in the bottom-right corner of Figure 5. Values on the right of gene ontology bar charts are log10(*P*-value) of corresponding ontology terms. Concise description of each gene set (G, H) is based on its ontology terms and is shown in bold font at the top of every gene set box.

Sets G and H of Figure 6 contain mRNAs whose levels during EGF stimulation are affected by the isogenic mutants studied, whether their expression in the WT is EGF-dependent (G) or EGF-insensitive (H), i.e. not responding to EGF stimulation. The initial levels of these mRNAs (at 0 min) are not significantly different in WT, PTEN KO and PIK3CA H1047R. Averaged profiles of mRNA clusters in both sets (Figure 7) reveal that this effect is much weaker than the constitutive effect of isogenic mutations (sets A–F, Figure 6), i.e. time courses of mRNA levels in PTEN KO and PIK3CA H1047R conditions are closer to WT. Interestingly, the mRNAs in these sets display a similar profile and are involved in the same biological processes as the mRNAs in sets 1 and 2 described in the analysis of WT versus A66 (Figure 3). mRNAs in sets 1 and G (EGF-dependent in WT) that show a peak of expression at early time points (0–90 min) code for proteins involved in transcription regulation and RNA synthesis. In the same sets, proteins encoded by mRNAs whose expression is maximum at later time points (180–300 min) are involved in RNA processing. Similarly, the proteins encoded by mRNAs in sets 2 and H (EGF-insensitive in WT) and overexpressed in PIK3CA H1047R are mainly involved in RNA metabolism and splicing. Proteins encoded by mRNAs that are overexpressed in PTEN KO play a role in cell respiration and energy derivation. Despite the functional similarities between the mRNAs regulated in the WT and in the mutant cell lines, one cluster in the set 2 (WT) is not present in set H. The proteins encoded by mRNAs in this cluster are linked to translation elongation and termination. This suggests that the chronic disruption of the PIP3 pathway, either via PTEN deletion or PIK3CA H1047R mutation, does not affect the regulation of those mRNAs.

#### Basal effects of the PI3K pathway mutations

mRNAs whose level is affected by the deletion of PTEN compared to WT but not by the constitutively active version of PI3Kα, regardless of EGF stimulation (comparison of WT versus PTEN KO at 0 min) are represented by sets E (EGF-dependent in WT) and F (EGF-insensitive in WT). Proteins encoded by the mRNAs present in the E cluster play a role in morphogenesis, development and proliferation. In contrast, proteins encoded by the mRNAs overexpressed in the F cluster are involved in cell respiration, energy derivation, same as mRNAs upregulated in PTEN KO in sets 2 and H, while the proteins encoded by the mRNAs underexpressed in the F cluster are involved in cell–cell adhesion.

mRNAs whose level is affected by the constitutively active version of PI3Kα compared to WT, but not by PTEN KO, regardless of EGF stimulation (comparison WT versus PIK3CA H1047R at 0 min) are represented by sets C (EGF-dependent in WT) and D (EGF-insensitive in WT). Proteins encoded by the mRNAs in set C, whose level reached a maximum after 180 min of EGF stimulation, are involved in RNA processing and splicing. Proteins encoded by mRNAs whose levels peaked earlier show an enrichment in generic GO terms related to cellular response to stimulus. However proteins encoded by mRNAs in set D, that are overexpressed in PIK3CA H1047R, play a role in mitosis. This biological function is specifically affected by the presence of the mutation PIK3CA H1047R, but not PTEN deletion and does not seem to be directly regulated via class I PI3K activity in the WT.

mRNAs in the sets A (EGF-dependent in WT) and B (EGF-insensitive in WT) are affected by both both PI3K H1047R and PTEN KO chronic effects. While genes encoding the mRNAs in set A are mainly involved in ‘dynamic’ development, locomotion and migration, those in set B contribute to the ‘static’ extracellular matrix/structure organization, biological cell adhesion, adenosine triphosphate synthesis and neutrophil aggregation.

### Control of mRNAs affected by PTEN KO and PIK3CA H1047R

mRNAs from sets A–H that were most significantly changed in mutant PTEN KO and PIK3CA H1047R cells were identified following the same procedure as for WT cells (Table 1). The majority of them are the same mRNAs already identified in WT cells. The mRNAs in Table 1 that uniquely belong to sets A-H correspond to TBP, EHF2, ZNF143, NRF1 and E2F1..5 motifs.

### Differential activity of the PRDM1 motif

**Figure 8.**
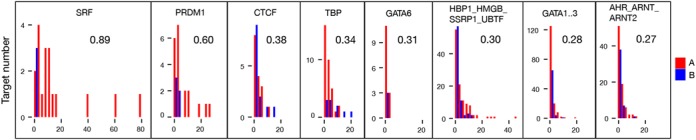
Distributions of scores S_*m*_ of gene sets A and B. Motifs are sorted by the degree of difference between set distributions of S_*m*_, namely by D statistic value of Kolmogorov–Smirnov test (see Materials and Methods).

Sets A (EGF-dependent in WT) and B (EGF-insensitive in WT) represent mRNAs that are strongly affected by both mutations. To identify potential differences in their transcriptional regulation we considered the distributions of S_*m*_ scores for each TFBM and tested them for equality using two different statistical tests (see Materials and Methods). The tests were in good agreement with each other and identified two main binding motifs, SRF and PRDM1 (Figure 8). Both motifs regulate genes in set A more strongly than in set B, suggesting that they are more active upon EGF stimulation. In agreement with previous results, increased activity of SRF is related to activation of the ERK pathway — one of the main pathways responding to EGF (all highly regulated SRF target genes from Figure 8 encode mRNAs found in Table 1). In contrast, the increase in PRDM1 activity in the presence of EGF and dependent on PIP3 signalling was not expected. Thus, we looked further at PRDM1 target genes that respond to EGF stimulation (red bars in Figure 8). Table 2 shows all 20 of these targets. Their functions were checked in the literature and Uniprot. We identified five genes in that group that may directly regulate the activity of the class I PI3K pathway (red colour in Table 2). Additionally, the VWA5A gene is a known breast tumour suppressor (green colour in Table 2). This suggests that there may be a transcriptional feedback loop in the class I PI3K signalling network which allows PIP3 to control the activity of its own pathway.

## DISCUSSION

In this study, we explored the consequences of various chronic and acute perturbations of PIP3 signalling (acute: p110α isoform-selective inhibitor A66 and EGF; chronic: H1047R-p110α mutation and PTEN deletion) on the mRNA landscape of MCF10a cells using mRNA-seq and bioinformatics.

We subdivided mRNAs into several groups based on their differential levels under different perturbations, and then clustered them according to their time courses. Subsequent GO enrichment analysis was performed on each mRNA cluster. In agreement with previously reported analysis of gene expression time courses (35) we observed that genes from the same cluster tended to regulate similar biological processes, whereas GO analysis of unclustered genes did not highlight any significant biological functions. In order to extract information on transcription factor activities possibly responsible for the differential effects of perturbations, we used the ISMARA tool (33). ISMARA has provided important insights into the structure of transcriptional networks in different systems and helped to predict novel regulatory circuits. ISMARA has been applied to both time-independent data, consisting of samples representing different experimental conditions (e.g. (36–38)), and time-dependent data, consisting of time-course samples (e.g. (39,40)). In addition, statistical methods were developed and applied to standard ISMARA output. Recently, a singular value decomposition (SVD) method was used to analyse TFBM activities provided by an ISMARA analysis of time-dependent data (41). It helped to successfully decompose several types of biological behaviour that corresponded to different experimental treatments. However, when we applied the same SVD method to TFBM activities obtained from ISMARA analysis of our data, we were unable to clearly interpret the biological meanings of the right singular vectors (data not shown). As a consequence, we used a more classical clustering approach (39) (Figure 4) which provided us with clearer biological insights. In turn, we also introduced a new approach for comparisons of the distributions of TFBM target scores (S_*m*_) using a non-parametric statistical test. It helped us to reveal a possible transcriptional feedback loop in PIP3 signalling.

### Mutations of PI3K and PTEN remodel the mRNA landscape

One of our main observations is that the effects of of PIK3CA-H1047R and PTEN deletion are dramatic, influencing the levels of thousands of mRNAs even in very closely related ‘isogenic’, unstimulated cells. This is entirely consistent with recent work that demonstrated major reshaping of the mRNA landscape by PIK3CA-H1047R in ‘isogenic’ cells related to those used in this study, an effect which the authors termed the ‘butterfly effect’ (22). However, we do not have parallel data from independent clones of these cell lines and therefore cannot define the extent to which these differences are a result of the specific genetic modifications versus chance events accumulated in the independent history of the cells. We compared our data with published data from two other labs for MCF10a cells (22) (GEO Accession number GSE63452) and (42) (Array Express accession number E-MTAB-2706), and with similar data for prostate epithelium (43) (Array Express accession number E-GEOD-47047). Interestingly, this revealed that although our data was globally highly similar to one study of MCF10a cells, it was more different from another study of MCF10a cells than it was from similar data from prostate epithelium cells. The pattern of differences may have been related to the conditions under which the cells were cultured. Indeed, the growing media contained cholera toxin ((22) and this study) or not (42), Charcoal dextran-treated FBS (42) or not ((22) and this study) and twice more EGF in (22,42) than in this study. The starvation medium of (22) and (42) still contained EGF (therefore desensitizing cells), and (22) used a different base medium (MCDB-170 instead of DMEM/F12). Finally (22) isolated the RNA at very different time after starvation (0–24 h instead of 0–3 h in this study). However, the importance of each of these factors is unclear. Further experiments should analyse independent, but ‘theoretically identical’ clones of ‘isogenic’ cell lines, treated according to the same protocol, to resolve this important issue.

### Most EGF effects on mRNA levels are PI3K-independent

The analysis of WT cell lines (Figure 3) also suggests that most of the EGF-induced changes in mRNA were due to PI3K-independent pathways (orange area of Venn diagram on Figure 3). This result was expected because EGF is known to activate several other important signalling pathways (44–48). The number of mRNAs changed by EGF and the scale of those changes were far greater than that induced by the so-called ‘butterfly effects’ (expression of H1047R-p110α or loss of PTEN). Figure 3 also revealed that the actual number of mRNAs whose expression is regulated by EGF via PI3K, i.e. when A66 counteracts the EGF effect in the WT, is more limited (groups 3 and 4). Further, the application of A66 to cells not stimulated with EGF revealed a number of mRNAs that were changed and that we classified as ‘sensitive to basal activity of PI3K’. The relatively limited effects of A66 either in the presence or absence of EGF are manifest in the very high similarity in the global mRNA landscapes of A66 treated and untreated cells (Figure 2A, WT versus A66). The acute nature of the perturbation on the wild-type cells compared to the chronic effects of H1047-p110α expression and PTEN deletion, with their associated ‘butterfly effects’, might yield a distinct cell phenotype. It will be important to extend this work by examining more prolonged periods of treatment with class IA PI3K inhibitors and the impact of such inhibitors in H1047R-p110α-expressing and PTEN deficient cells.

### Different mutations trigger different ‘Butterfly effects’

We attempted to predict the possible phenotypic consequences of the changes in mRNA levels. When our analyses started with the entire family of mRNAs changed by perturbation of PIP3 signalling there was little clear evidence of GO term enrichment. This is probably to be expected when a central signalling pathway, that controls and integrates many aspects of cell behaviour, is modulated. Therefore, we split the data into various groups.

The largest effect of PTEN deletion was an upregulation of gene expression. These genes are generally involved in cell respiration and energy derivation (∼600 genes in Figure 6, group F and ∼900 genes in Figure 7 group H). This finding is in agreement with previously reported studies of PTEN deletion in other cell types (49,50). Another interesting effect of PTEN deletion was a unique fraction of mRNAs whose levels dropped dramatically in PTEN deleted cells compared to WT in the absence of EGF (Figure 5A, asymmetric distribution of blue points around the black line). Further GO enrichment analysis revealed that mRNAs that are ‘shut down’ are mainly involved in cell proliferation, cell–cell adhesion and signalling (Figure 6, groups E and F).

Changes in mRNA levels in PIK3CA-H1047R-expressing cells in the absence of EGF are more symmetrical than those of PTEN KO cells (Figure 5B — symmetric distribution of green points around black line). About 200 mRNAs whose levels are increased in H1047R-p110α-expressing cells but do not respond to EGF (Figure 6, group D) mainly encode for proteins involved in mitosis. In contrast, the ∼300 mRNAs that are increased by expression of H1047R-p110α and respond to EGF (Figure 6, group C) encode for proteins involved in RNA processing and splicing. Interestingly, a large number (∼900, Figure 7, group H) of mRNAs which do not respond to EGF stimulation but are more weakly increased by expression of H1047R-p110α were also involved in RNA processing and splicing.

Overall, the proteins encoded by the mRNAs that responded to EGF have essentially ‘dynamic’ cell functions (development, locomotion, migration, wound healing, morphogenesis, see Figure 6, groups A and E), while the mRNAs that did not respond to EGF have more ‘static’ functions (extracellular matrix, cell adhesion, see Figure 6, D, B and F). This effect was particularly prominent for the mRNAs from groups A and B in which the levels of mRNAs were affected by both mutations.

**Figure 9.**
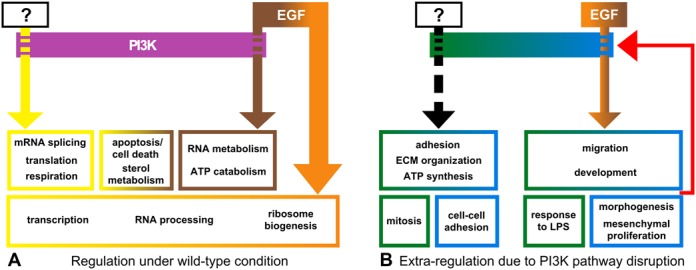
A concise diagram of all discovered properties of PI3K signalling pathway. (**A**) Properties of WT cell lines; (**B**) Properties of mutant cell lines.

Based on the analysis of all expression profile clusters in WT and mutant cell lines we created a concise diagram of PIP3 signalling mapping out the manner in which the analysis objectively resolved our data (Figure 9).

### Differential activation of TF binding sites reveal a transcriptional feedback loop

We attempted to determine whether some of the changes in mRNA landscape induced by manipulation of PIP3 signalling could be due to changed transcription. This approach had the potential to identify transcription factors like FOXO-family members regulated by PIP3. We used ISMARA to identify those transcription factor binding motifs whose activity changed most with perturbation of PIP3 signalling. This process identified a number of TFBMs including those for PRDM1 and SRF (see below). Motifs binding proteins of the FOXO-family were identified but with much lower strength. In particular, we looked at the activity of transcription binding sites responsible for the differential effect of EGF present in both mutations. Differential analysis of the two sets A and B using ISMARA identified two TFBMs — PRDM1 (binding site for the transcriptional repressor PRDM1, also known as BLIMP1) and SRE (binding site for SRF) — that were mainly responsible for the effect of mutations on mRNAs from set A (responding to EGF in WT, Figure 8) but not the mRNAs from set B (not responding to EGF in WT). When we looked at the mRNAs from set A that are potentially regulated by PRDM1 (Table 2), we noticed that several target genes of the PRDM1 motif could affect PIP3 pathways (CYLD, PIK3IP1, NEDD4L, LPXN, GAB2). This suggests that a PRDM1-PIP3 transcriptional feedback loop is present in the system. However, no direct regulation of PRDM1 has been documented as far as we know. There are two possibilities: (i) a direct feedback loop through activation of PRDM1 by PI3K; (ii) an indirect activation of PRDM1 by other pathways responding to EGF and whose function is affected by PI3K pathways. PRDM1 is barely expressed in the absence of EGF. EGF treatments increased its expression five-fold in the WT and both mutant cell lines (Supplementary Figure S3). This increase peaked at 40 min and disappeared at 100 min. Conversely, the default ‘activity’ of the PRDM1 binding motif is high until 180 min, after which it sharply drops. Since PRDM1 is a transcriptional repressor, this is consistent with a fast effect of EGF increasing its (PRDM1) transcription followed by a repression of its targets. Studying the other target genes might also reveal new regulation of PI3K pathways. For instance VWA5A encodes the von Willebrand factor A domain containing 5A, a known breast tumour suppressor (51).

### Overlapping effects of several perturbations correlate with PIP3 signalling logic

**Figure 10.**
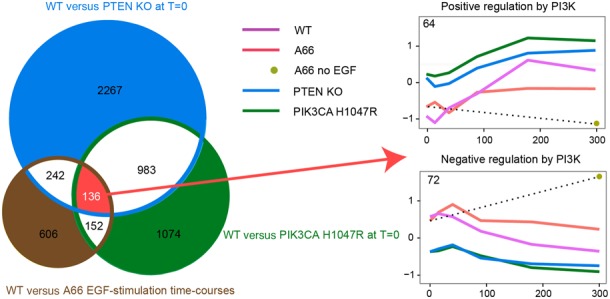
mRNAs regulated in-keeping with the logic of PIP3 signalling (see text for description). The Venn diagram represents an overlap of three gene sets identified by differential expression analysis (see captions for details). Gene expression time course profiles represent the average of many genes (exact number is shown in each profile).

In this study we attempted to combine a number of temporally and mechanistically distinct PIP3 perturbations to reveal mRNA changes more likely to be directly controlled by PIP3 signals amongst the very large number of changes resulting from indirect homeostatic effects. The number of mRNAs that were influenced by all the PIP3 perturbations employed was far smaller (136). Importantly, the very large majority of these mRNAs were regulated in-keeping with the logic of PIP3 signalling i.e. mRNA levels increased by loss of PTEN and expression of H1047R-p110α in both basal and EGF-stimulated conditions, but were reduced by A66; or *vice versa*. This pattern contrasted with that seen in the lists of mRNAs regulated by fewer of the PIP3 perturbations, where other perturbations induced changes in a given mRNA that appeared inconsistent with the logic of PIP3 signalling. This observation suggests the 136 mRNAs regulated by all PIP3 perturbations are likely to be more direct targets of PIP3 signalling. Clustering the group of 136 mRNAs via the kinetics of their responses resolved two stable groups (Supplementary Table S3 and Figure 10), the average profiles of which showed either; (i) an increase in mRNA levels in alignment with the logic of increased PI3K/PIP3 signalling (*n* = 64), or, (ii) a decrease in mRNA levels in alignment with the logic of decreased PI3K/PIP3 signalling (*n* = 72).

### Are changes at the level of mRNAs reflected in protein amounts?

Several proteomics datasets are available for MCF10a cell line (22,52,53). Unfortunately, in all those studies experimental conditions differed from our experiment. For instance in (52,53) the same medium was used to grow the cells, however the cells were not starved in serum before the protein extraction. We also looked at the PRoteomics IDEntifications (PRIDE) database and found three studies on MCF10a cells. Similarly, in the most relevant study (accession number PXD000599) the cells were not starved and the focus of the study was on the expression of phosphorylated proteins only. Additionally, in all these studies, only a few (usually one) or different (from ours) data points of EGF stimulation were considered. Based on our observation that slight differences in experimental conditions can have even more dramatic effects on gene expression than genetic mutations (see Results, Mutations of PI3K and PTEN remodel the mRNA landscape), it is difficult to directly meaningfully compare those datasets to our results.

By using several bioinformatics approaches this study provides a valuable insight into understanding chronic and acute effects of the PI3K signalling pathway on the genome wide transcriptome. Our findings confirm the concept of the ‘butterfly effect’, in which thousands of mRNAs are changed as a result of single oncogenic mutations of the PI3K/PIP3 pathway being introduced into cells. We were able to identify a relatively small number of mRNAs (∼130) that appear to be the primary targets of PIP3-signalling within this huge wave of changes. Some of these PIP3-sensitive changes in mRNA appear to be driven by PIP3-regulation of the PRDM1 transcription factor binding motif. A potential transcriptional feedback loop by which PIP3 signalling may control the class I PI3K signalling network has also been identified.

## Supplementary Material

SUPPLEMENTARY DATA
