# Supplementary material for: Perturbations of PIP3 signalling trigger a global remodelling of mRNA landscape and reveal a transcriptional feedback loop
Source: Nucleic Acids Res. 2015 Oct 12;43(20):9663–79. doi: 10.1093/nar/gkv1015 (PMC4787766; doi:10.1093/nar/gkv1015)
Supplement: SUPPLEMENTARY DATA [file supp_43_20_9663__index.html]

Perturbations of PIP3 signalling trigger a global remodelling of mRNA landscape and reveal a transcriptional feedback loop — SUPPLEMENTARY DATA 

# Perturbations of PIP3 signalling trigger a global remodelling of mRNA landscape and reveal a transcriptional feedback loop

## SUPPLEMENTARY DATA

- SUPPLEMENTARY DATA
