# Supplementary material for: Perturbations of PIP3 signalling trigger a global remodelling of mRNA landscape and reveal a transcriptional feedback loop
Source: Nucleic Acids Res. 2015 Oct 12;43(20):9663–79. doi: 10.1093/nar/gkv1015 (PMC4787766; doi:10.1093/nar/gkv1015)
Supplement: SUPPLEMENTARY DATA [file supp_gkv1015_nar-02096-n-2015-File013.pdf]

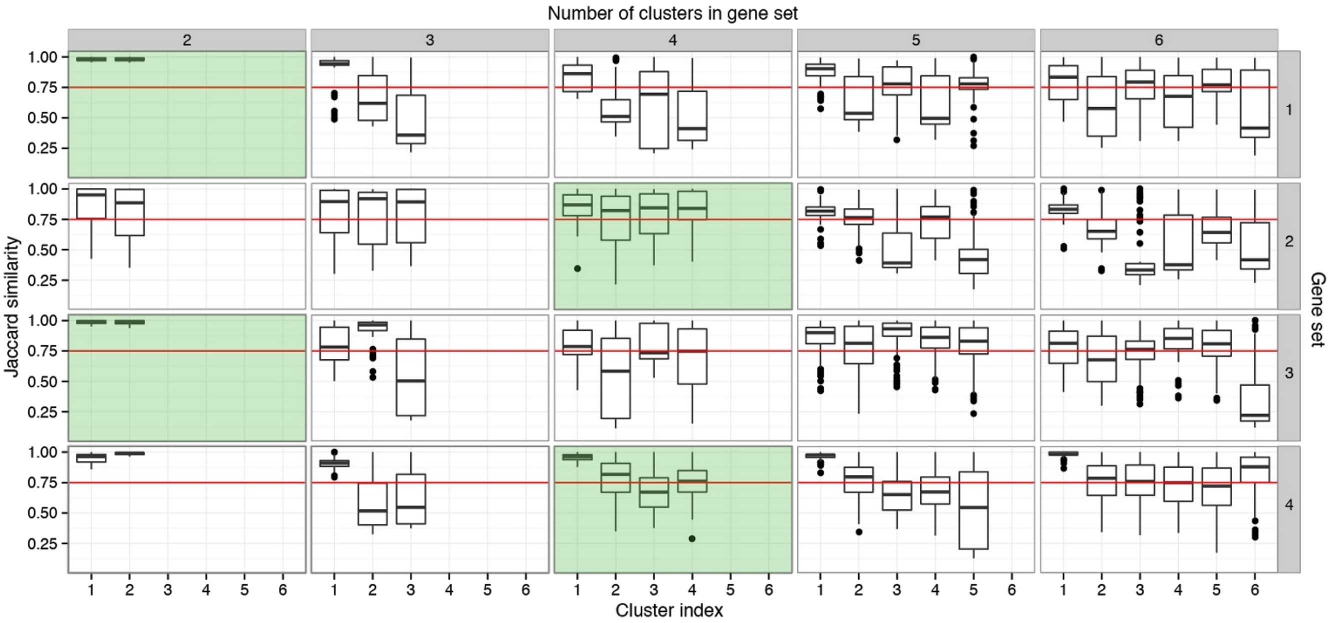

Figure S1. Boxplots of Jaccard similarities of clusters identified using 100 bootstrappings of gene sets 1-4. The upper and lower "hinges" correspond to the first and third quartiles (the 25th and 75th percentiles). The upper whisker extends from the hinge to the highest value that is within 1.5*IQR of the hinge, where IQR is the inter-quartile range, or distance between the first and third quartiles. The lower whisker extends from the hinge to the lowest value within 1.5*IQR of the hinge. Data beyond the end of the whiskers are outliers and plotted as points. Clusterings coloured with light green were selected for further analysis.

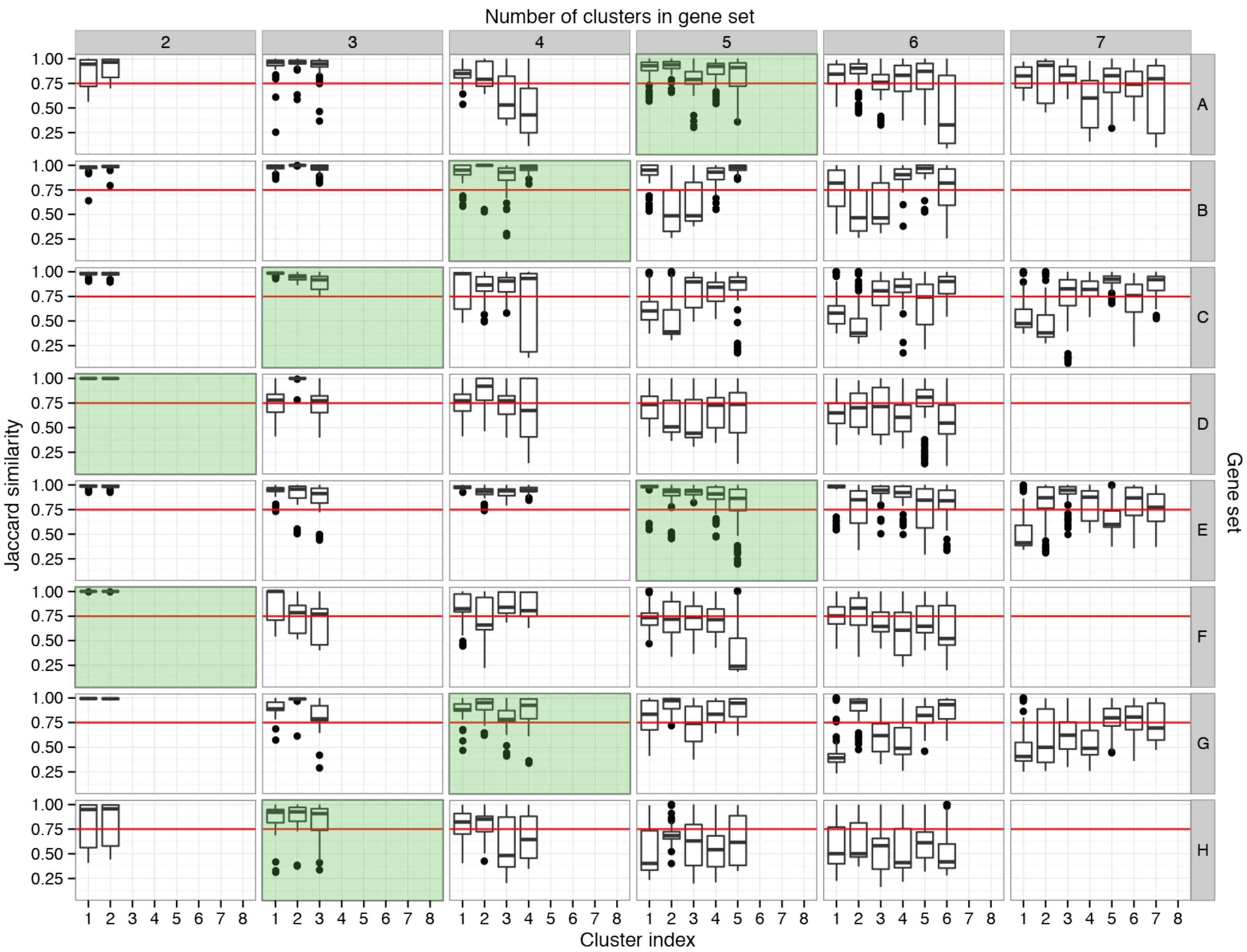

Figure S2. Boxplots of Jaccard similarities of clusters identified using 100 bootstrappings of gene sets A-H. The upper and lower "hinges" correspond to the first and third quartiles (the 25th and 75th percentiles). The upper whisker extends from the hinge to the highest value that is within 1.5*IQR of the hinge, where IQR is the inter-quartile range, or distance between the first and third quartiles. The lower whisker extends from the hinge to the lowest value within 1.5*IQR of the hinge. Data beyond the end of the whiskers are outliers and plotted as points. Clusterings coloured with light green were selected for further analysis.

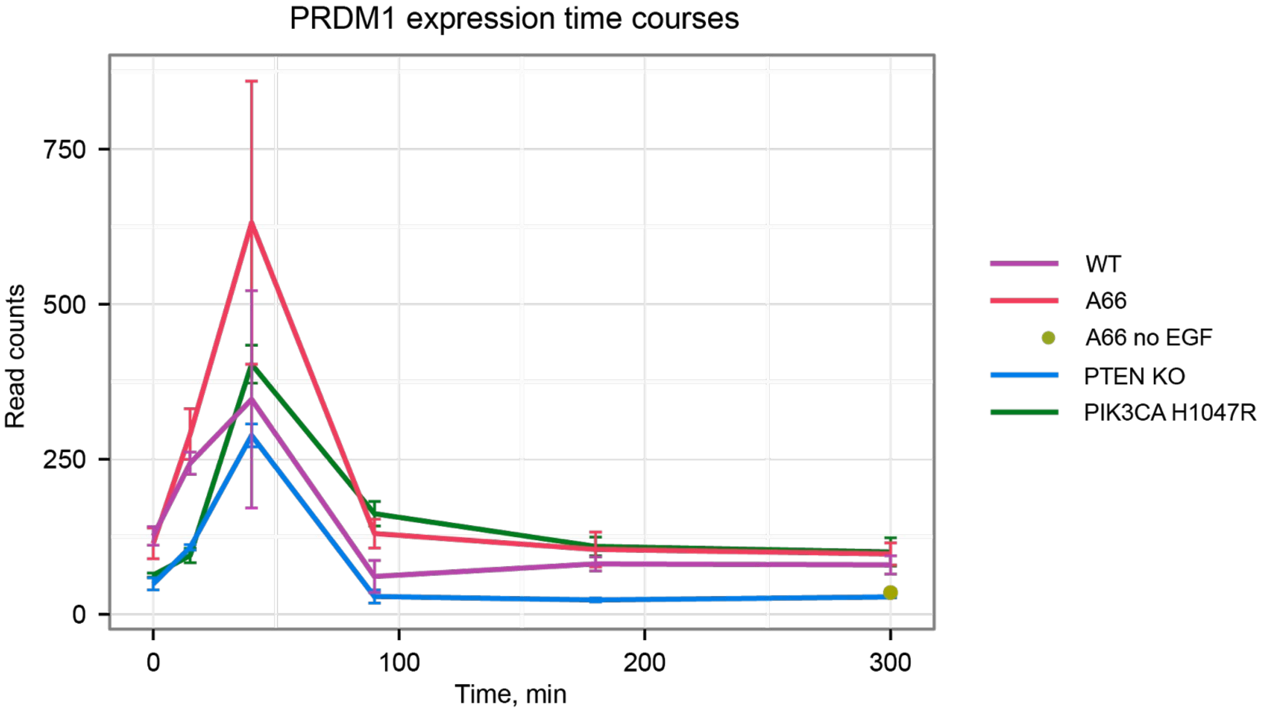

Figure S3. PRDM1 expression profile over the time course of EGF stimulation.

TF binding motif	KS statistics	p-value
SRF.p3	0.89	0.015
PRDM1.p3	0.60	0.085
CTCF.p2	0.38	0.191
TBP.p2	0.34	0.266
GATA6.p2	0.31	0.929
HBP1_HMGB_SSRP1_UBTF.p2	0.30	0.001
GATA1..3.p2	0.28	0.001
AHR_ARNT_ARNT2.p2	0.27	0.022
EHF.p2	0.26	0.079
ATF5_CREB3.p2	0.26	0.073
ZNF384.p2	0.24	0.016
bHLH_family.p2	0.21	0.222
ZNF143.p2	0.21	0.366
TFAP2B.p2	0.19	0.017
E2F1..5.p2	0.19	0.625
UUUUUGC	0.18	0.234
KLF12.p2	0.18	0.210
NFKB1_REL_RELA.p2	0.17	0.318
NRF1.p2	0.17	0.724
GTF2I.p2	0.15	0.129
YY1.p2	0.14	0.251
ARNT_ARNT2_BHLHB2_MAX_MYC_USF1.p2	0.14	0.904
TFDP1.p2	0.13	0.100
TGIF1.p2	0.13	0.982
HIC1.p2	0.12	0.163
RREB1.p2	0.11	0.576
SP1.p2	0.08	0.488
KLF4.p3	0.07	0.620

Supplementary Table1. Comparison of two distributions of Sm using the non-parametric Kolmogorov-Smirnov test.

TF binding motif	MW statistics	p-value
GATA6.p2	20	0.953
SRF.p3	52	0.006
PRDM1.p3	77	0.071
TBP.p2	118	0.151
CTCF.p2	139	0.132
TGIF1.p2	313	0.873
E2F1..5.p2	388	0.577
ARNT_ARNT2_BHLHB2_MAX_MYC_USF1.p2	503	0.521
NRF1.p2	713	0.904
ZNF143.p2	760	0.469
EHF.p2	800	0.053
bHLH_family.p2	1639	0.053
ATF5_CREB3.p2	1640	0.155
UUUUUGC	1673	0.199
NFKB1_REL_RELA.p2	2214	0.266
AHR_ARNT_ARNT2.p2	2324	0.021
KLF12.p2	3516	0.066
RREB1.p2	3731	0.295
ZNF384.p2	4727	0.001
YY1.p2	4919	0.190
HBP1_HMGB_SSRP1_UBTF.p2	5646	0.000
GTF2I.p2	7006	0.071
GATA1..3.p2	7216	0.000
TFAP2B.p2	10947	0.006
HIC1.p2	16782	0.571
TFDP1.p2	18315	0.094
KLF4.p3	24221.5	0.497
SP1.p2	31602.5	0.128

Supplementary table 2. Comparison of two distributions of Sm using the non-parametric Mann-Whitney test.

a) **b)**

Ensembl gene ID	HGNC symbol
ENSG00000003436	TFPI
ENSG00000030110	BAK1
ENSG00000049283	EPN3
ENSG00000051596	THOC3
ENSG00000063660	GPC1
ENSG00000069011	PITX1
ENSG00000077238	IL4R
ENSG00000099624	ATP5D
ENSG00000099821	POLRMT
ENSG00000103326	CAPN15
ENSG00000104885	DOT1L
ENSG00000104899	AMH
ENSG00000104980	TIMM44
ENSG00000104998	IL27RA
ENSG00000105447	GRWD1
ENSG00000106012	IQCE
ENSG00000109321	AREG
ENSG00000110047	EHD1
ENSG00000110987	BCL7A
ENSG00000112715	VEGFA
ENSG00000123977	DAW1
ENSG00000126790	L3HYPDH
ENSG00000128228	SDF2L1
ENSG00000129932	DOHH
ENSG00000130312	MRPL34
ENSG00000130821	SLC6A8
ENSG00000134013	LOXL2
ENSG00000135334	AKIRIN2
ENSG00000140905	GCSH
ENSG00000145555	MYO10
ENSG00000148426	PROSER2
ENSG00000153885	KCTD15
ENSG00000155846	PPARGC1B
ENSG00000158321	AUTS2
ENSG00000159166	LAD1
ENSG00000160293	VAV2
ENSG00000161847	RAVER1
ENSG00000164086	DUSP7
ENSG00000165655	ZNF503

Ensembl gene ID	HGNC symbol
ENSG00000004799	PDK4
ENSG00000012822	CALCOCO1
ENSG00000013588	GPRC5A
ENSG00000033327	GAB2
ENSG00000054793	ATP9A
ENSG00000069712	KIAA1107
ENSG00000070731	ST6GALNAC2
ENSG00000071242	RPS6KA2
ENSG00000073282	TP63
ENSG00000074054	CLASP1
ENSG00000074527	NTN4
ENSG00000089327	FXYD5
ENSG00000090863	GLG1
ENSG00000100100	PIK3IP1
ENSG00000102760	RGCC
ENSG00000110756	HPS5
ENSG00000112182	BACH2
ENSG00000115112	TFCP2L1
ENSG00000116285	ERRFI1
ENSG00000118515	SGK1
ENSG00000121879	PIK3CA
ENSG00000122042	UBL3
ENSG00000122557	HERPUD2
ENSG00000124098	FAM210B
ENSG00000128591	FLNC
ENSG00000128833	MYO5C
ENSG00000133639	BTG1
ENSG00000133789	SWAP70
ENSG00000135821	GLUL
ENSG00000136155	SCEL
ENSG00000136653	RASSF5
ENSG00000137193	PIM1
ENSG00000137831	UACA
ENSG00000138433	CIR1
ENSG00000138764	CCNG2
ENSG00000139263	LRIG3
ENSG00000143515	ATP8B2
ENSG00000143850	PLEKHA6
ENSG00000144802	NFKBIZ

ENSG00000168040	FADD
ENSG00000169230	PRELID1
ENSG00000169972	PUSL1
ENSG00000170684	ZNF296
ENSG00000175315	CST6
ENSG00000175591	P2RY2
ENSG00000175606	TMEM70
ENSG00000177971	IMP3
ENSG00000178726	THBD
ENSG00000179041	RRS1
ENSG00000179820	MYADM
ENSG00000181649	PHLDA2
ENSG00000182768	NGRN
ENSG00000184564	SLITRK6
ENSG00000186395	KRT10
ENSG00000188042	ARL4C
ENSG00000188483	IER5L
ENSG00000196182	STK40
ENSG00000203499	FAM83H-AS1
ENSG00000225210	AL589743.1
ENSG00000226085	UQCRFS1P1
ENSG00000247626	MARS2
ENSG00000261221	ZNF865
ENSG00000261373	VPS9D1-AS1
ENSG00000264281	CTD-2031P19.4

ENSG00000144834	TAGLN3
ENSG00000149212	SESN3
ENSG00000151690	MFSD6
ENSG00000153071	DAB2
ENSG00000155893	ACPL2
ENSG00000158445	KCNB1
ENSG00000160179	ABCG1
ENSG00000163347	CLDN1
ENSG00000163453	IGFBP7
ENSG00000163898	LIPH
ENSG00000164379	FOXQ1
ENSG00000164615	CAMLG
ENSG00000164649	CDCA7L
ENSG00000164733	CTSB
ENSG00000165312	OTUD1
ENSG00000166689	PLEKHA7
ENSG00000167601	AXL
ENSG00000169248	CXCL11
ENSG00000169398	PTK2
ENSG00000171517	LPAR3
ENSG00000173706	HEG1
ENSG00000175318	GRAMD2
ENSG00000176641	RNF152
ENSG00000181104	F2R
ENSG00000185920	PTCH1
ENSG00000185950	IRS2
ENSG00000196730	DAPK1
ENSG00000197375	SLC22A5
ENSG00000198189	HSD17B11
ENSG00000213626	LBH
ENSG00000214944	ARHGEF28
ENSG00000223573	TINCR
ENSG00000237973	hsa-mir-6723

Supplementary table 3. List of genes which mRNA levels change upon PIP3 perturbations in correlation with the PIP3 signalling logic. a) increase in mRNA levels. b) decrease in mRNA levels.
